# The first complete mitogenomes and phylogeny of Georgian Mountain Cattle

**DOI:** 10.1080/23802359.2022.2110531

**Published:** 2022-08-24

**Authors:** N. Kunelauri, M. Gogniashvili, V. Tabidze, G. Basiladze, I. Cardinali, H. Lancioni, T. Beridze

**Affiliations:** a Institute of Molecular Genetics, Agricultural University of Georgia, Tbilisi, Georgia; b Agricultural University of Georgia, Tbilisi, Georgia; c Department of Chemistry, Biology and Biotechnology, University of Perugia, Perugia, Italy

**Keywords:** Georgian Mountain Cattle, complete mitochondrial DNA, single nucleotide polymorphism, mitogenome variation, *Bos taurus* phylogeny

## Abstract

The Georgian Mountain Cattle (GMC) (Species: *Bos taurus* Linnaeus, 1758 – aurochs, domesticated cattle, domestic cattle (feral), Aurochs, Subspecies: *Bos taurus taurus* Linnaeus, 1758) is a local breed from Georgia. It is well adapted to the harsh mountain conditions of the Caucasus, resistant to different pathogens and able to provide milk during the lowest feed rations. In this study, we report for the first time the complete mitochondrial genome sequence of GMC. We selected the five unique control region mitochondrial haplotypes of GMC and sequenced for the complete mitogenome, using Illumina MiSeq platform. The results of our research showed a total of 59 substitutions and seven indels, in comparison to the Bovine Reference Sequence (BRS; V00654), with 15 SNPs never observed before. The complete mitochondrial DNA (mtDNA) phylogenetic analyses revealed hitherto unknown haplotypes falling outside the known taurine diversity. Four mitogenomes fell within haplogroup T (sub-lineages T1, T3, and T5), while one belonged to haplogroup Q (branch Q1). The combination of our results with precision agriculture holds great promises for the identification of genetic variants economically affecting important traits of GMC cattle.

Historians recognized several places of agricultural and animal domestication and one of them is located at the border of the prehistoric Georgian tribe’s resettlement in South-West Asia. In addition, the International Union for Conservation of Nature included the biological species common in the Caucasus in the list of the most diverse and endangered hotspots (http://biodiversity-georgia.net/index.php?pageid=905). Over the centuries, local strains of Georgian cattle evolved by the joint action of environmental conditions and human mediated selection for unique agricultural properties. Among these groups, the Georgian Mountain Cattle (GMC) represents an important local breed, endemic to the Caucasus. In 1980, about 80,000 heads were registered, but population data are not updated since 2006 when it counted less than 30,000 individuals, thus highlighting a trend that is strongly decreasing. GMC is a very small-sized breed, but it has a unique productivity potential, both for meat and milk. The peculiarities of GMC compared to other cattle breeds are the good adaptation to the harsh climatic conditions of Caucasus and to poor impoverished food, endurance, and sustenance. Moreover, this breed seems to be a variety of the Ancient Georgian cattle, but its exact origin is still unknown (www.fao.org/dad-is/).

Our previous research conducted on the most variable region of the mitochondrial DNA (mtDNA) (D-loop) revealed five novel haplotypes, never observed in any other cattle breed (Kunelauri et al. 2019). The GMC mitogenomes were phylogenetically grouped in the T lineage close to the Near Eastern Cattle as well as to the European Red Mountain Cattle (Di Lorenzo et al. 2016; Ludwig et al. 2016). To further investigate the GMC phylogeny, we selected five samples and characterized their complete mitogenomes for the first time.

Blood samples were collected from Dusheti District (Georgia) (42° 5′ 0″ N, 44° 42′ 0″ E) and DNA was extracted as previously described (Kunelauri et al. 2019). DNA Blood samples (accession/voucher numbers: LC576822/0025, LC580279/0010, LC597385/0002, LC597384/GE10-67557, and LC597386/GE10-67700) were preserved in a tissue collection in the Institute of Molecular Genetics of Agricultural University of Georgia (Contact person: n.kunelauri@agruni.edu.ge).

Experiments were carried out according to the norms of the International Animal Care and Use Committee (IACUC) and Bioethics Committee of the Free University of Tbilisi (Georgia). Genomic DNA libraries were constructed using NEBNext Ultra DNA Library Prep Kit (New England Biolabs, Ipswich, MA) and sequenced on the Illumina MiSeq platform (San Diego, CA). Reads were assembled using the SOAPdenovo2 software program. The complete mtDNA sequences (five samples) were deposited in GenBank and mapped against the Bovine Reference Sequence (BRS) (GenBank accession no. V00654). The entire mitogenome included one control region (D-loop), 13 protein-coding genes, two rRNA genes, and 22 tRNA genes, thus reflecting the typical mitochondrial genomes of cattle (Hiendleder et al. 2008).

In addition to the D-loop already explored, we have identified other variable regions and a total of 15 unique SNPs, never registered before. Overall, we detected 49 transitions and 10 transversions (18 substitutions fell in the D-loop region, 32 in the CDS, three tRNA, and six rRNA, whereof A169G, G9682C, and A13310C were shared by all haplotypes), and seven indels. Twenty-nine mutations occurred as singletons.

The phylogenetic analysis was performed by comparing GMC mitogenomes with all mtDNAs of other cattle breeds available from GenBank. The resulting maximum-likelihood phylogenetic tree is presented in Figure 1 and shows the peculiar position of our GMC mitogenomes within the cattle worldwide phylogeny, revealing that four mitogenomes fell within haplogroup T (sub-lineages T1, T3, and T5), while one belonged to haplogroup Q (branch Q1), closely related to other sequences derived from various breeds, but all suitable for mountain grazing (Olivieri et al. 2015). Our previous investigation already detected a high incidence of the mtDNA haplogroup Q in GMC that might confirm an ancient origin of this lineage in the region that represents an important migration route through the Caucasus. Recent studies, focusing on plant domestication in the same area, found wild predecessors of five Georgian endemic wheat subspecies in Fertile Crescent, quite far from the South Caucasus (Beridze 2019). One hypothesis to explain what is called the ‘Wheat Puzzle’ is that speakers of Protogeorgian language could be separated from Protoeurasiatic language speakers after migration from Africa to the Arabian Peninsula and later moved to the northern part of Mesopotamia, where wheat was domesticated. Then, Georgian speakers could have migrated further to South Caucasus (notably Georgia) and might have brought the domesticated wheat subspecies as well as their cattle, thus explaining the presence of ancient mtDNA lineages in this area.

**Figure 1.**
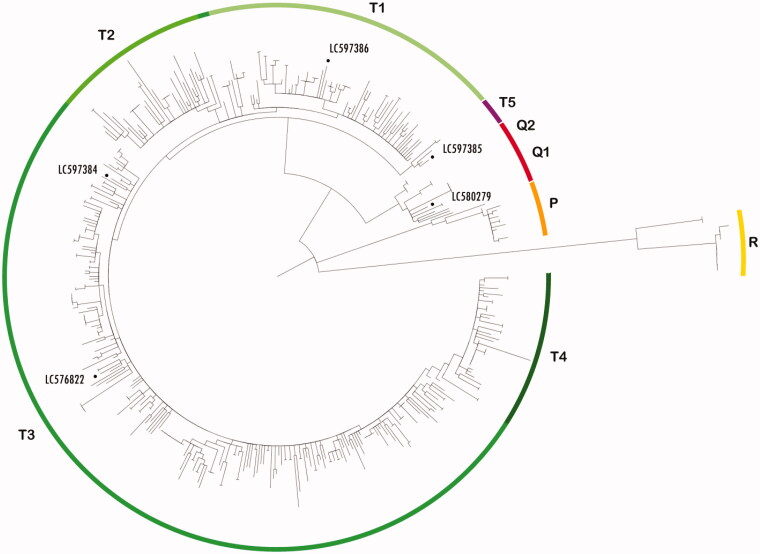
Phylogenetic tree of cattle mitogenomes. The phylogenetic relationships between mtDNA sequences of Georgian Mountain Cattle with accession numbers LC576822; LC580279; LC597384; LC597385 and LC597386 (highlighted by black dots) and other 445 cattle obtained from NCBI were analyzed using Maximum-Likelihood Sequences within haplogroup I were excluded from the representation; T1, T2, T3,T4, T5, P, Q1, Q2 and R haplogroups were reported.

In conclusion, we provide for the first time five unique mitogenomes of GMC, and in addition to the already published control region mtDNA substitutions, we identify an even higher mitochondrial variation of GMC.

## Data Availability

The genome sequence data that support the findings of this study are openly available in GenBank of NCBI at https://www.ncbi.nlm.nih.gov/ under the accession nos. LC576822, LC580279, LC597384, LC597385, and LC597386. The associated BioProject, SRA, and Bio-Sample numbers are PRJNA814276, SRS12233564, SRS12233565, SRS12233566, SRS12233567, SRS12233568 and SAMN26542468, SAMN26542469, SAMN26541585, SAMN26542510, SAMN26542519, respectively.
